# Application of acid activated natural clays for improving quality of Niger (Guizotia abyssinica Cass) oil

**DOI:** 10.1016/j.heliyon.2022.e09241

**Published:** 2022-04-06

**Authors:** Jemal Mohammed Yassin, Yoseph Shiferaw, Abebe Tedla

**Affiliations:** a Department of Chemistry, College of Natural and Computational Science, Debre Berhan University, Debre Berhan, P. O. Box. 445, Ethiopia; b Department of Chemistry, College of Natural and Computational Science, Dambi Dollo University, Dambi Dollo, P. O. Box. 260, Ethiopia

**Keywords:** Color, Adsorption, Natural clays, Edible oil, Acid activation

## Abstract

Natural clay has been considered as one of the most appropriate, locally available, effective, and low-cost edible oil adsorptive purifying materials. The removal of impurities and colored substances from edible oil increases the quality of oils. This study reports the application of acid activated different clays for bleaching of Niger oil. The clay samples were collected from different parts of North Shoa Zone, Ethiopia namely Zemero, Seladengay and Mehal Meda, and treated by three different acid concentrations (15 %, 20 % and 25 %) with HCl, HNO_3,_ and H_2_SO_4_. The acid activation boosts the behavior of the clays by manipulating its physical and chemical properties, which highly responsible for the removal of impurities. The characteristic of the clay samples were characterized by using X-ray diffraction (XRD), X-ray Fluorescence spectrometry (XRF), Fourier transform infrared (FTIR) and UV-vis spectrophotometer, respectively. The Niger oil (Guizotia abyssinica Cass) was treated with each activated clays to evaluate for their use as local adsorptive materials. The results indicated that all the clay samples activated with H_2_SO_4_ demonstrated the highest bleaching efficiency compared to the clays activated with HCl and HNO_3_ under similar conditions. Therefore, 25 % sulfuric acid activated Zemero clay is the most efficient (94.5 %) with adsorbent dose (1 g), contact time (30 min), and temperature (90 °C) compared to Seladengay and Mehal Meda clay samples. The results indicate the adsorption capacities of all the three activated clays and their potential applications for efficient treatment and purification of oils to improve flavor, taste, and shelf life of oil products.

## 1 Introduction

Niger (Guizotia abyssinica Cass) is one of the five Guizotia species which has originated from the Ethiopian highlands and distributed to other countries. This plant produces oilseed, which is locally known as noog/nug, Nyjer, ramtil or ramtilla, inga seed and black seed in different parts of Ethiopia (Getinet and Sharma, 1996). Edible oil is mostly used for cooking purpose and extracted from the seed of Niger plant. Niger seed oil has high nutritional values (Griffiths, 1990) since contains linoleic acid as the primary fatty acid (75–80 %), followed by palmitic and stearic acids (7–8 %) and oleic acid (5–8 %). In addition to the above valuable nutrients, the oil may include impurities like gums, wax, trace metals, peroxides, phosphatides, and pigments, which can degrade the oil's quality (Ramadan and Morsel, 2002). The removal of such impurities helps improve the flavor, taste, shelf life, appearance, and sensory quality of the final oil products, which increases customer acceptance (Nguetnkam et al., 2008; Farihahusnah et al., 2011).

Bleaching edible oil removes coloring pigments (generated by carotenoids, chlorophyll and xanthophylls), peroxides, and other impurities that cause poor quality and instability in edible oils. It is normally accomplished by treating crude or refined oil with suitable absorbent. Physical adsorption because of van der Waals interaction, chemical bonding because of covalent and ionic interaction, ion exchange, molecular trapping and chemical decomposition are the adsorption mechanisms that occur in the bleaching process of oil (Noyan et al., 2007). Natural clay minerals have drawn attention among low-cost adsorbents on account of their easy and abundant availability, as well as high adsorption capabilities for cations and polar molecules (Yang et al., 2006; Monvisade and Siriphannon, 2009; Yassin, 2018). Among various types of clay materials, bentonite is a kinds of clay that is widely used in the oil industries, both in its natural and activated forms. Activated bentonite is considered as ideal clay material owing to its higher bleaching efficiency. Clays may be treated in a variety of ways to improve their performance to bleach and remove impurities. Among the techniques, the most commonly accepted method in edible oil industries is acid treatment. Acid concentration, treatment duration, temperature, and adsorbent dosage are some of the parameters to consider while optimizing the bleaching effectiveness of clays (Monvisade and Siriphannon, 2009).

A multitude of surface features of clays, including as structure, ion exchange capacity, specific surface area, mechanical and chemical stability, water holding capacity, and reactivity, have a substantial impact on the physical and chemical properties of clays. Because of their surface chemistry, clay materials have different adsorption processes and mechanisms. Even though it is recommended to utilize clays as they are, chemical alteration to change their surface properties to boost their adsorption capacity is widespread (Liang et al., 2013). Numerous investigation have been conducted on variety of clay materials found in various countries for their potential application in bleaching, cleaning, and other applications due to its surface properties (Komadel et al., 1990; Christidis et al., 1997; Kirali and Lacin, 2006; Novakovic et al., 2008; Rozic et al., 2010; Foletto et al., 2011; Ajemba and Onukwuli, 2012; Shiferaw et al., 2019). Although clay minerals are abundantly available in large quantities in Ethiopia, North Shoa Zone, particularly in Zemero, Seladengay, and Mehal Meda areas, to the best of our knowledge, no work has been reported regarding the chemical composition and oil bleaching capacity of these clays. We expected that these materials might have great adsorption capacities with better performance and could be a source of bleaching materials for different oil industries. Therefore in this paper we present the chemical compositions of the clays and their adsorptive purification capacity on Niger oil after chemical modification of their surface properties with different acids.

## 2 Material and methods

### 2.1 Chemicals

Analytical grade chemicals and reagents were used without any further purification. Nitric acid (HNO_3_, >69 %, from Breckland Scientific Supplies), Sulfuric acid (H_2_SO_4_, >98 %, from Breckland Scientific Supplies), Hydrochloric acid (HCl, > 37 %, from Sigma-Aldrich), and Acetone (CH_3_COCH_3_, >99 %, from Sigma-Aldrich) were used in this study.

### 2.2 Sample collection and preparation

The clay samples were collected from different places of North Shoa Zone (Mehal Meda, Seladengay, and Zemero), Amhara region, Ethiopia. The Niger oilseed was obtained from a local market. Each clay samples were ground and washed with distilled water to remove impurities. The mixtures were stirred and the slurries were then decanted leaving behind impurities such as sand and stones. The clay samples were dried in the oven at 105 °C for 8 h and sieved separately through a 350 μm mesh sieve.

### 2.3 Preparation of activated clays

Three different concentrations (15 %, 20 %, and 25 %) of Sulfuric acid (H_2_SO_4_), Hydrochloric acid (HCl) and Nitric acid (HNO_3_) were used to treat the natural clays. 10 g of each clay samples (Zemero, Mehal Meda, and Seladengay) was separately mixed with 100 mL of 15 %, 20 % and 25 % HNO_3_, H_2_SO_4_, and HCl in a conical flask, and the mixture was refluxed at 90 °C for 30 min with continuous stirring. The refluxed samples were cooled, filtered under vacuum pump and washed repeatedly with distilled water until the filtrate was free of ions. The filtered samples were dried at 105 °C for 2 h. All the acid activated clay samples were again crushed down to a particle size in a way that would pass through a 350 μm mesh size and then kept in separate polyethylene bags for further use.

### 2.4 Characterization methods

The chemical composition was determined by using PAN alytical Cubix XRF simultaneous X-ray Spectrometry. The mineralogical compositions of the non-activated and acid activated clays were recorded by X-ray diffraction (XRD) using a Shimadzu, XRD-7000 X-ray diffractometer equipped with a Cu-Kα X-ray radiation (λ = 1.5418Å). The surface functional group of the non-activated and activated clay samples and the bleached and unbleached Niger oil were recorded by FTIR (PERKIN ELMER). UV-Visible spectrophotometer (SP65) was used to measure the absorption of the bleached and unbleached oils.

### 2.5 Optimization of parameters

To determine the best bleaching conditions, four parameters (temperature, contact time, clay dose and acid concentration) were optimized in the following ways.

**Temperature:** The effect of temperature was examined by making contact time and activated clay dose constant while varying the temperature (50, 70, 90 and 100 °C). A mixture of Niger oil (25 g) and activated clays (2 g) was heated at 50, 70, 90 and 100 °C while keeping other parameters constant to determine the best temperature at which the bleaching efficiency is maximum. This experiment was done for all activated clay samples following the same procedure. The absorbance was measured using the UV-Visible spectrometer, and then the bleaching efficiency was calculated.

**Contact time:** temperature and dose of activated clay was made constant while varying the contact time (10, 20, 30 and 40 min) to determine the optimum contact time. A mixture of Niger oil (25 g) and activated clays (2 g) was heated at optimum temperature by making other parameters constant to determine the best time for maximum bleaching. The same experiment was done for all activated clay samples. The bleached oil was cooled and centrifuged. The absorbance was measured using the UV-Visible spectrometer, and the best bleaching efficiency was determined.

**Clay dosage:** The effect of clay dose was examined by using the optimum temperature and contact time, while varying the dosage (1, 1.5 and 2 g). To determine the best dosage of clay in which the bleaching efficiency is high, a mixture of Niger oil (25 g) and activated clays (1, 1.5 and 2 g) was heated at optimum temperature while keeping other parameters fixed. The same experiment was done for all activated clays.

**Acid concentration (acid/clay ratio):** The effect of acid concentration was determined by varying the concentration of HNO_3_, H_2_SO_4_, and HCl (15 %, 20 %, and 25 %) while keeping temperature at 90 °C, dose of clay (1 g), Niger oil (25 g) and contact time (30 min). The absorbance was measured using the UV-Visible spectrometer, and the bleaching efficiency was calculated. The optimum amount of activated clay that caused maximum bleaching efficiency was identified.

The absorbance was measured using the UV-Visible spectrometer, and the bleaching efficiency was calculated. Then bleaching efficiency was determined using the following Eq. (1):

(1) $$\text{Bleaching ​Efficiency ​}\left(\text{\%}\right)=\frac{\left(\text{Co}-\text{C}\right)}{\text{Co ​}}\times 100$$

Where Co, and C are absorbance of unbleached and bleached oils respectively. Maximum absorption wavelength (λ_max_) of unbleached Niger oil was determined by UV-Vis spectrophotometer through scanning from 200 nm to 800 nm with 20 nm wavelength interval. The λ_max_ was 450 nm as presented in Figure 7b.

## 3 Results and discussion

### 3.1 XRF characterization of clay samples

The chemical compositions of clay minerals were determined by X-ray fluoresce spectrometry. The XRF result shows that the major oxides present in the clay samples are SiO_2_, Al_2_O_3_, Na_2_O, K_2_O, MgO, CaO, CaCO_3_, SO_3_, and Fe_2_O_3,_ but silica and alumina are the dominant oxides as indicated in Table 1. In activated clays from Zemero, Mehal Meda, and Seladengay, the compositions of SiO_2_ and Al_2_O_3_ are found to be 51.62 %, 45.98 %, 40.38 %; and 12.89 %, 17.17 %, 21.62 % respectively. Moreover, the higher content of SiO_2_/Al_2_O_3_ ratio (greater than 2) shows an excess of SiO_2_ in the form of quartz, as well as the existence of octahedral substitution, which reveals the dominance of montmorillonite charged clay type (ratio 2:1). The chemical composition analysis has demonstrated that Zemero clay consists of higher silica content compared to other clays, which may be explained by the depletion of the interlayer and octahedral cations, implying that it has a great potential for bleaching oils as well as for production of floor tiles (Alcàntara et al., 2008).

**Table 1:** Chemical Composition of Zemero, Mehal Meda, and Seladengay clays activated with H_2_SO_4_ (25 %).

Composition	Zemero (%)	Mehal Meda (%)	Seladengay (%)
Al_2_O_3_	12.899	17.171	21.616
Fe_2_O_3_	2.544	6.259	5.727
MgO	0.520	0.848	0.475
CaO	2.411	1.325	0.634
CaCO_3_	4.305	2.366	1.132
K_2_O	0.705	0.782	0.715
SiO_2_	51.621	45.977	40.376
SO_3_	0.214	0.198	0.144
Na_2_O	0.314	0.455	0.195

### 3.2 XRD characterization

**Figure 1:**
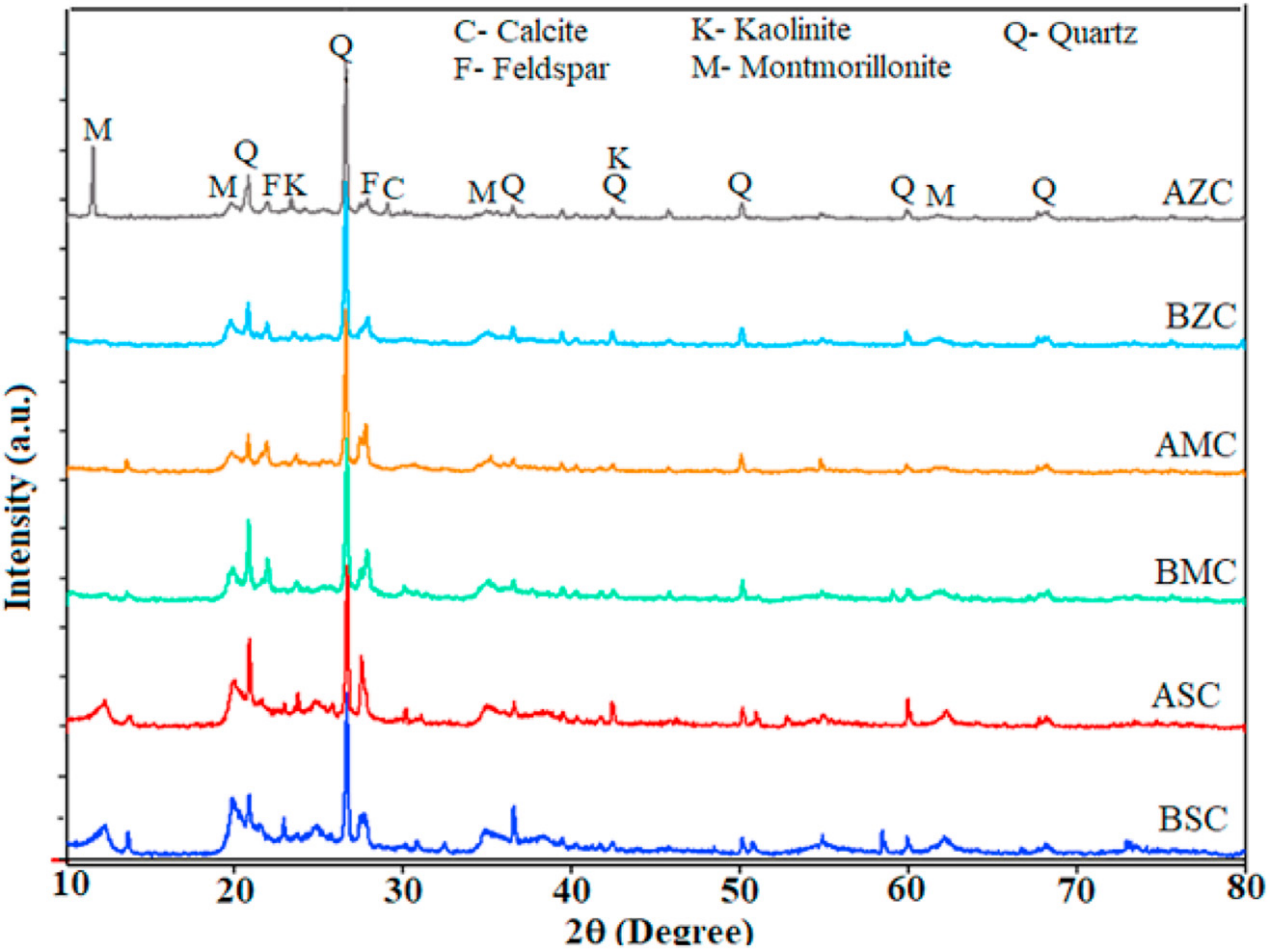
The XRD pattern of Seladengay, Mehal Meda and Zemero natural clay before and after activation with 25% H_2_SO_4_ (AZC: Zemero Clay after activation; BZC: Zemero Clay before activation; AMC: Mahel Meda Clay after activation; BMC: Mahel Meda Clay before activation; ASC: Seladengay Clay after activation; BSC: Seladengay Clay before activation).

XRD measurement was also performed to further compare the mineralogical composition of samples after and before activation using X-Ray diffractometer, equipped with Cu-Kα radiation (40 kV, 30 mA, λ = 0.15405 nm). The XRD patterns of samples (Seladengay, Mehal Meda, and Zemero clays) before and after acid activation are depicted in Figure 1. Accordingly, the XRD pattern at 2θ values of 11.61^o^, 13.62^o^, 19.84^o^, 35.03^o^ and 61.76^o^ correspond to montmorillonite [JCPDS card file No.003-0016], kaolinite [96-900-9235] (2θ ≈ 23. 41^o^ and 42.50^o^), quartz [96-901-3322/2601] and JCPDS Card file No. 01-079-1910 (2θ ≈ 20.88^o^, 26.63^o^, 36.57^o^, 42.50^o^, 50.13^o^, 60.00^o^ and 68.21^o^), calcite [96-101-0929] (2θ ≈ 29.09^o^), and feldspar (2θ ≈ 21.88^o^ and 27.82^o^) (Krishna and Susmita, 2007; Zorica et al., 2011; Fethi et al., 2015). In each sample, the deposit phase contains more than one phase in various amount, with the most dominant mineral appeared at 26.63^o^ (2θ), which corresponds to the quartz phase as the major one at 0.334 nm. In addition, a small amount of minerals such as feldspar and calcite are also found. The presence of montmorillonite, which is recognized as the most prevalent clay mineral in acid activated Zemero and Seladengay clays (before and after acid activation), is likely indicated by the occurrence of the diffraction peak at 2θ ≈ 11.61^o^ (7.57Å) which is consistent with reported literature (Krishna and Susmita, 2007; Amari et al., 2018).

### 3.3 FTIR characterization of clay samples

**Figure 2:**
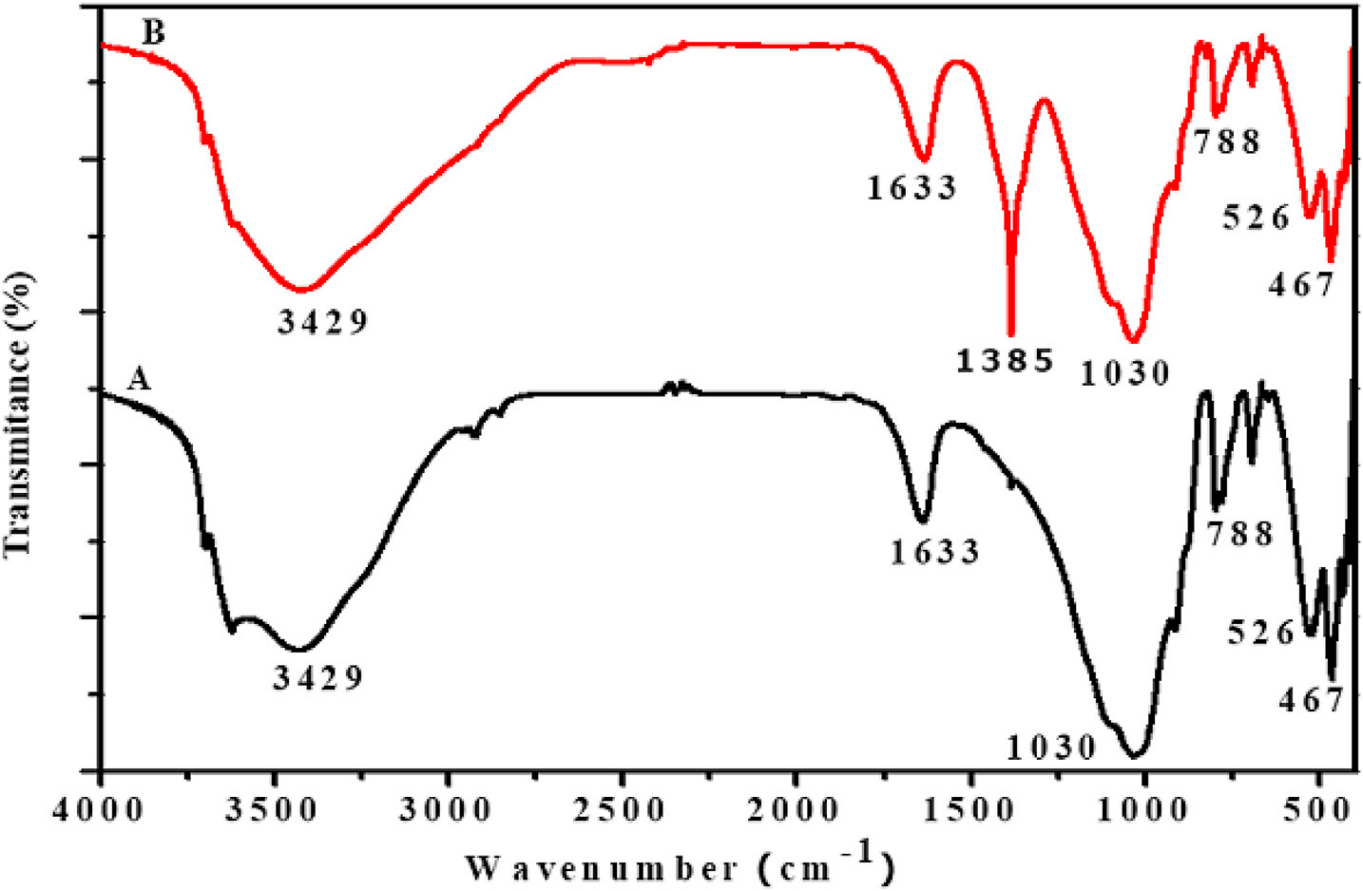
The FTIR spectra of (A) Zemero clay before activation and (B) after activation with 25 % sulfuric acid.

**Figure 3:**
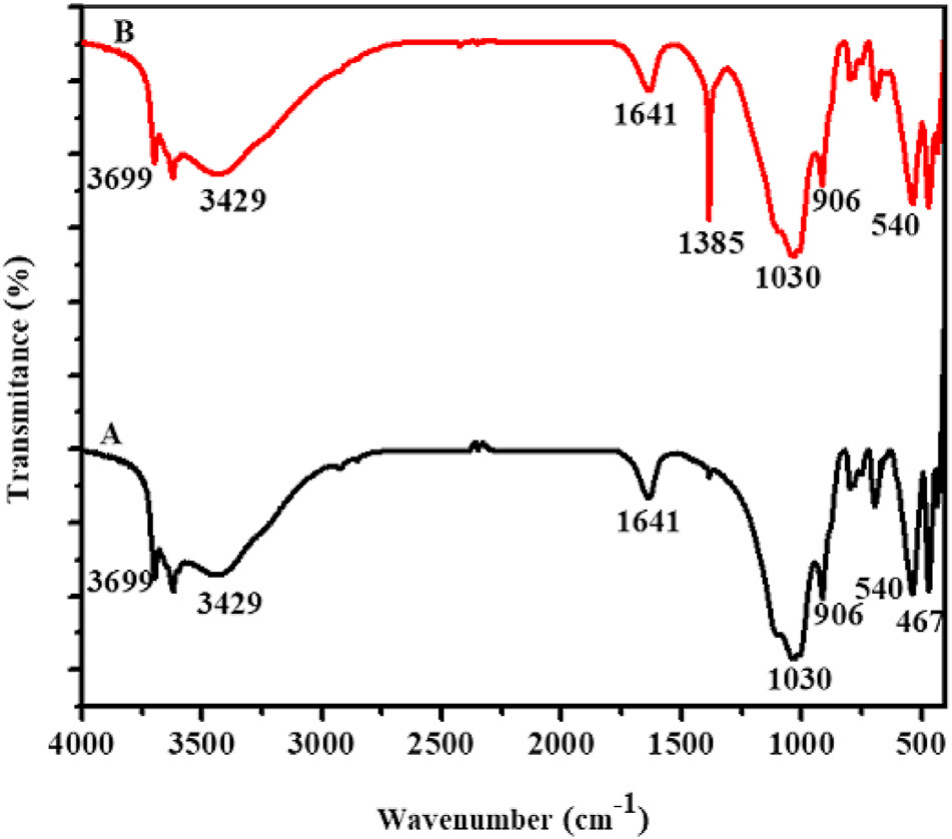
The FTIR spectra of (A) Seladengay clay before activation and (B) after activation with 25% sulfuric acid.

**Figure 4:**
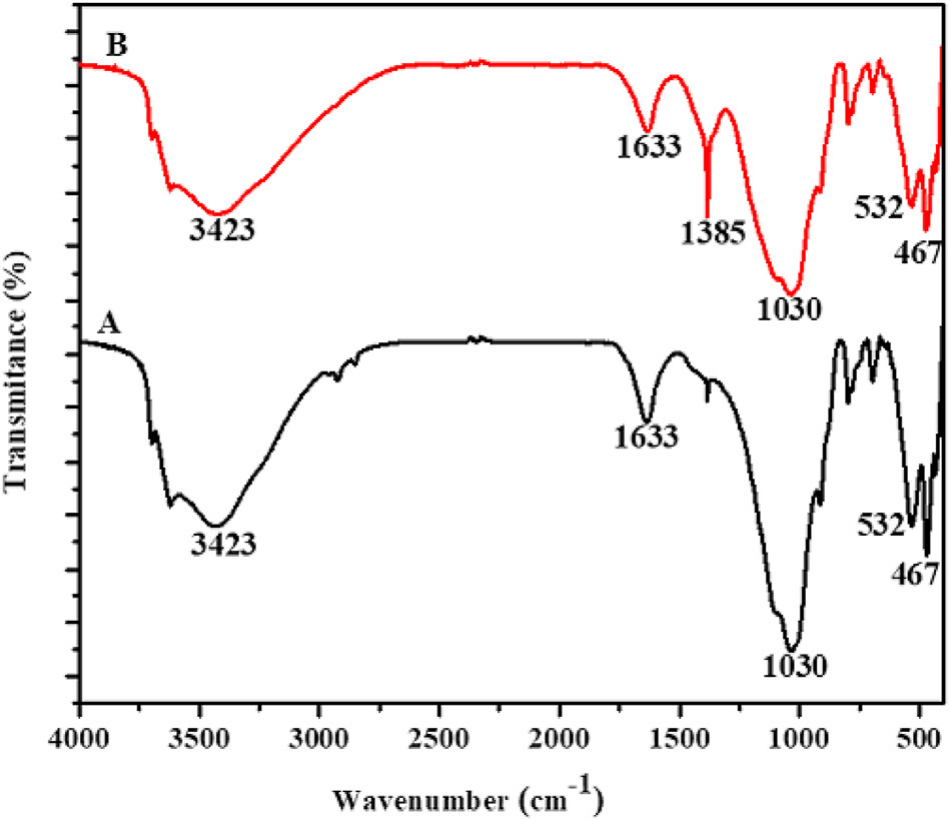
The FTIR spectra of (A) Mehal Meda clay before activation and (B) after activation with 25% sulfuric acid.

After optimization, highest bleaching efficiency was observed for those clays activated with sulfuric acid (25 %) at temperature 90 °C, dose of clay (1 g), contact time (30 min) and for Niger oil (25 g). Figures 2, 3, and 4 shows the FTIR spectra of Zemero, Seladengay and Mehal Meda clay samples before and after activation with 25 % sulfuric acid, respectively. During the activation of the clay samples, protons from the sulfuric acid penetrate into the clay structures and attacking the OH groups, causing the alteration of the absorption bands ascribed to the OH vibrations and octahedral cations (Pentrák et al., 2018). The absorption bands observed at 3429, 1633, 1385, 1030, 788, 526 and 467; 3699, 3620, 3429, 1641, 1385, 1030, 906, 690, 540 and 460 cm^−1^, and 3423, 1633, 1385, 1030, 532, and 467 cm^−1^ corresponds to Zemero, Seladengay and Mehal Meda activated and non-activated clay samples respectively.

The bands at 3699 and 3620 cm^−1^ is due to the stretching vibration of the OH group of the water molecule. The stretching bands’ intensities at 3429 and 3423 cm^−1^ are attributed to O-H stretching for free Si-OH in almost all natural hydrous silicates, revealing the montmorillonite characteristic band (Felhi et al., 2008). Moreover absorption bands observed at 1633 and 1641 are associated with H-O-H bending vibration in the clay. On the other hand, the bands at 526, 532, 540 and 690 cm^−1^ are caused by Si-O-Al vibrations in the clay samples (Boey et al., 2011). The intensity of FTIR absorption bands at 1385 cm^−1^ increased after activation of the clays due to carbonate stretching of calcite (Kloprogge et al., 2002; Majid, 2011, 2012). However, no significant change in band intensity was observed at 1030 cm^−1^ (Si-O stretching), suggesting that the original clay structure has not been completely destroyed. The absorption band observed at 467 cm^−1^ the Si-O-Si associated with bending vibration which is consistent with previously reported results (Boey et al., 2011).

### 3.4 Bleaching studies of Niger oil

There are several factors that influence the adsorption capacity of activated clay and its performance of removing color and impurities from oils. Some of the most important ones are concentration of activating agent, temperature, contact time, clay dosage, and clay quality (Díaz and Santos, 2001). It is of paramount importance to determine the optimum condition that offers highest bleaching efficiency to remove coloring and other impurities from oil.

**Figure 5:**
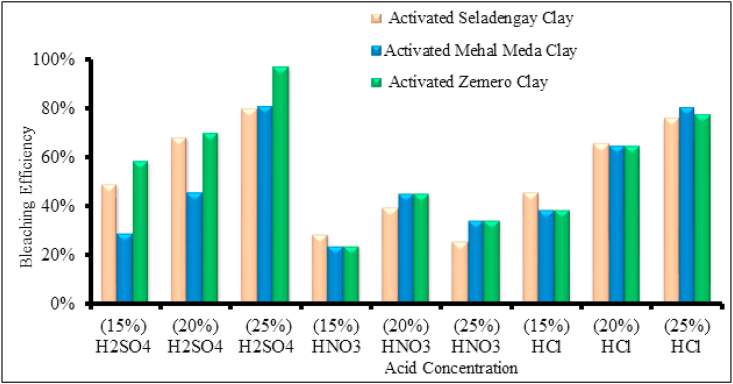
Activated Seladengay, Mehal Meda, and Zemero clay bleaching performance with different acids.

Concentration of acid and type of acids are one of the important factors that affect the bleaching efficiency. The UV-Vis absorbance of bleached and unbleached oil were measured, and the bleaching efficiency of each of the activated clays were calculated using Eq. (1). Figure 5 shows the bleaching efficiencies of the three activated clays with three different acids. In general the sulfuric acid treated clays appeared to be a more superior acid by showing the best result in dissolving octahedral cations and demonstrated higher bleaching efficiencies than those activated with HNO_3_ and HCl activated clays. And no significant variation in decolorizing performance was observed for the clays treated with HCl and HNO_3_. However, bleaching efficiency differences were noted at the three concentrations of H_2_SO_4_. For instance, Mehal Meda and Seladengay activated clay at concentration (25 %) with HCl and H_2_SO_4_ showed almost similar bleaching efficiency as indicated in Figure 5.

On the other hand, the decolorizing performance of activated Zemero clays was more effective than the two activated clays (Seladengay and Mehal Meda) which might be due to its high silica content (Table 1) and the greater effect of the acid attack on its structure. Because hydrated silica plays a crucial role in oil bleaching, the bleaching effectiveness of clay improves as the silica concentration of the clay increases (Komadel, 2016). The higher the silica content in the activated clay the greater the ability of the material to adsorb colored and other impurities in oils (Boey et al., 2011; Acquah et al., 2016). Furthermore, it was observed that, with the exception of HNO_3_, bleaching efficiencies increased as the concentration of the acids increased up to 25 %. For all activated clays utilized in this study, the maximum bleaching performance is at 20 % acid content, as shown in Figure 5, while non-activated clays have poor bleaching performance (not included). This phenomenon is quite different from the two acids. It is noted that an increase in acid concentration leads to improvement in physicochemical properties of the clay which may be due to substitution of cations including K^+^, Na^+^, and Ca^2+^ in octahedral and tetrahedral sites with H^+^ ions, and Al^3+^, Fe^3+^, and Mg^2+^ ions are released from both sites. This exposes the outer surface of the clay particles. However the decolorizing efficiency of the clays started to decline beyond 20 % for HNO_3_ and 25% for H_2_SO_4_ and HCl activation. Futher treatment of the clay with greater acid concentration causes leaching of Al^3+^, Fe^3+^, and Mg^2+^ from the octahedral positions that results in destruction of crystal structure of clay which in turn causes the decease in surface area (Ajemba and Onukwuli, 2012; Komadel, 2016).

**Figure 6:**
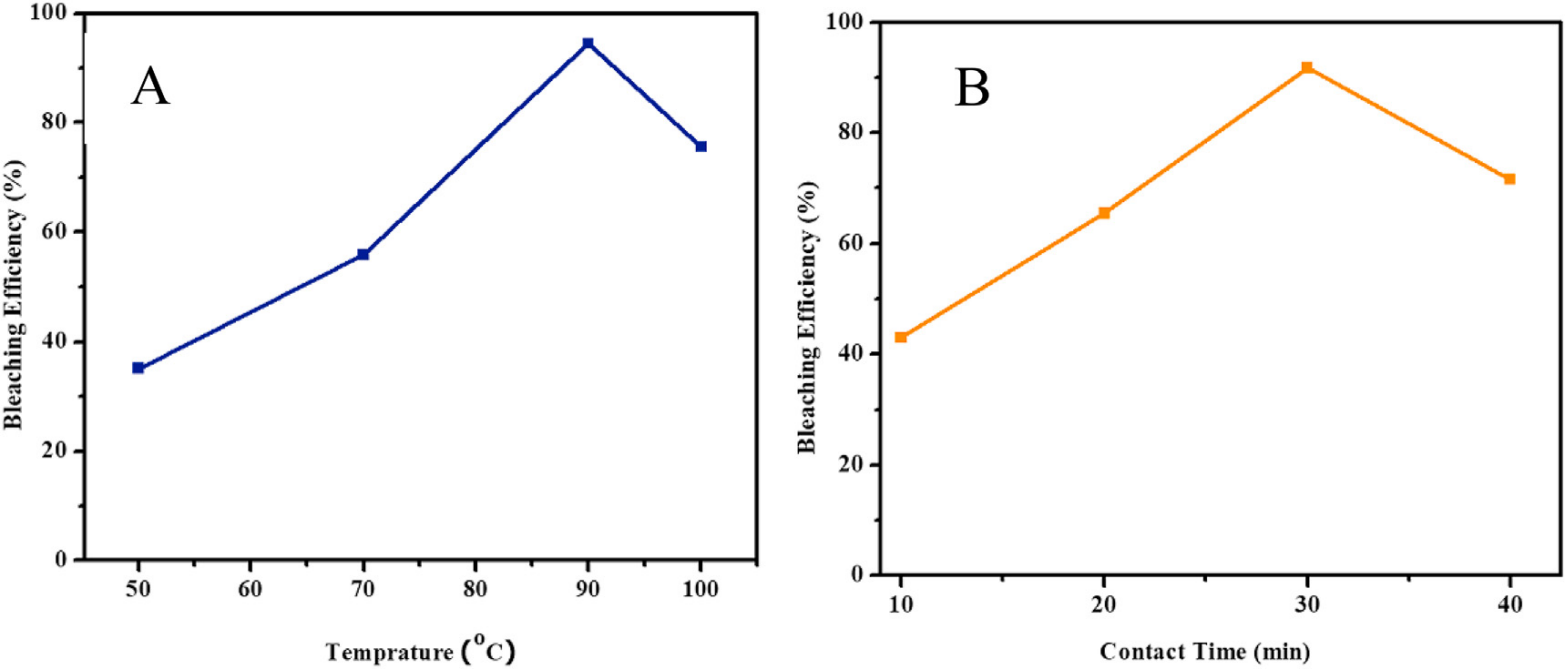
(A) Temperature and (B) contact time effect on bleaching Niger Oil.

Figure 6a indicates the effect of temperature on efficiency of activated Zemero clay to adsorb pigments and coloring compounds from Niger oil. Five different temperatures were considered in this study (60, 70, 80, 90 and 100 °C). The result showed that the bleaching efficiency increased as the temperature increased, where the maximum bleaching efficiency (94.5 %) was achieved at 90 °C and started to drop with further increase in temperature. This could be due to undesirable structural changes of the oil molecules via oxidative degradation and isomerization. The oil viscosity decreased with increasing temperature up to 90 °C resulting in a better dispersion of particles which improves clay-oil interactions, and flowability.

As indicated in Figure 6b, the bleaching efficiency of the activated clay increases with increasing contact time, suggesting that the activated clay has a capacity to remove pigments and other impurities with in short times. The efficiency of removing color and other impurities rapidly increased up to 30 min of contact time, when maximum performance (91.8 %) was recorded, before declining. The increase of bleaching efficiency with contact time can be explained by the fact that there are initially a significant number of vacant surface sites are available for removal of color and impurities but after certain time the vacant surface site can be occupied and get exhausted.

**Figure 7:**
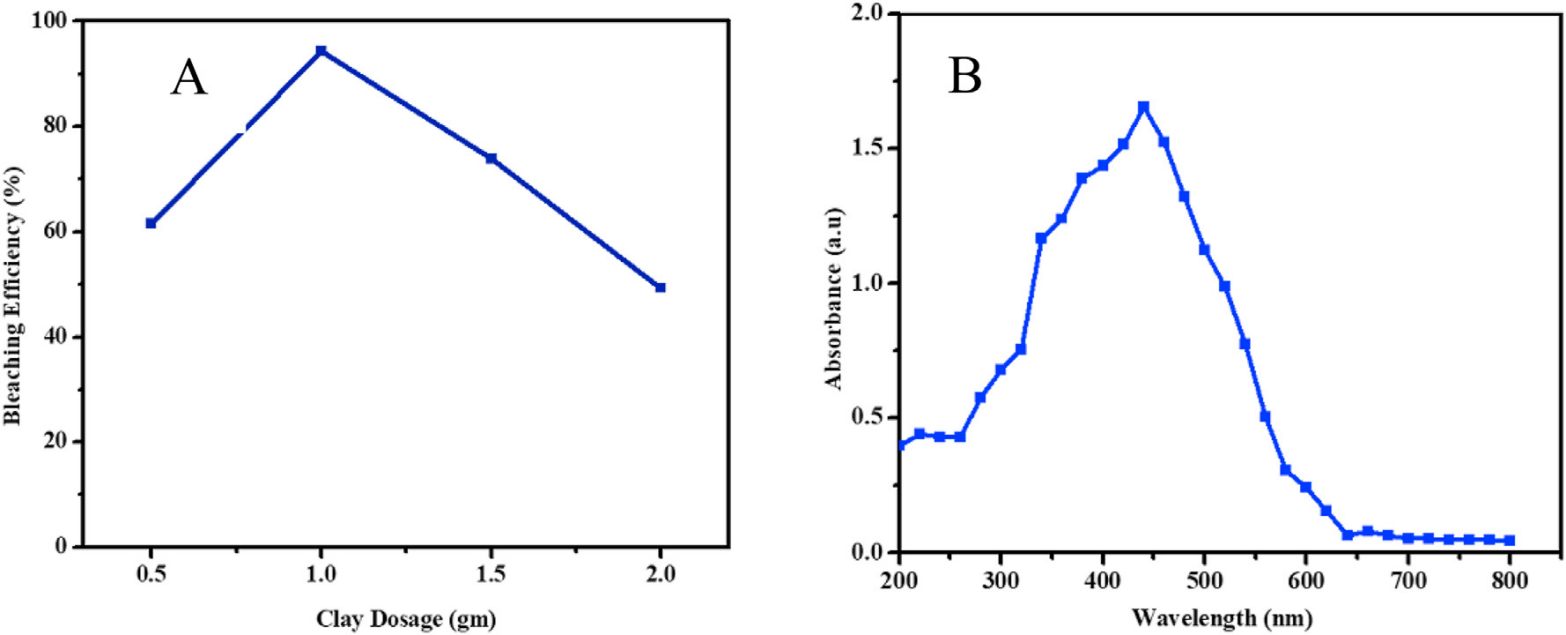
(A) The effect of clay dosage on bleaching Niger Oil, (B) UV-Vis absorbance spectrum of unbleached Niger Oil.

Figure 7a shows the relationship between amount clay and the bleaching efficiency, whereas Figure 7b displays the UV-Vis absorbance spectrum of unbleached Niger Oil. The performance of decoloring of the oil increased with the amount of clay up to 1 g and then deteriorated when the dosage increased beyond 1 g, indicating that 1 g is an optimum amount to have highest bleaching efficiency. The increase in bleaching efficiency with increasing dosage is due to the number of active sites available for adsorption increase. Once the bleaching efficiency at equilibrium between the adsorbent/oil mixtures has reached, no further pigment removal by the excess adsorbent dosage added.

**Figure 8:**
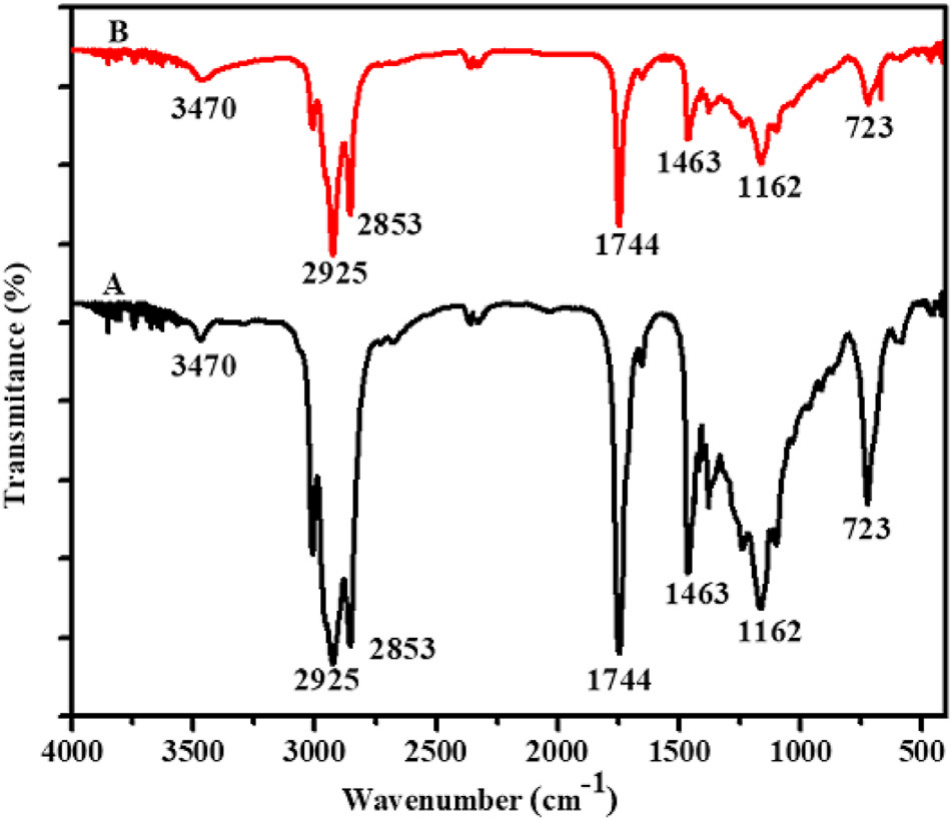
The FTIR spectra of (A) before bleaching and (B) after bleaching of Niger Oil with activated Zemero clay.

For comparison purpose, the FTIR spectra of Niger oil before and after treatment with acid-activated Zemero clay was measured (Figure 8). Absorption bands observed at 3010, 2925, 2853, 1744, 1463, 1162 and 723 cm^−1^ in both treated and untreated Niger oils. The absorbance peaks at 3010, 2925 and 2853 cm^−1^ are due to asymmetric and symmetric CH_2_ stretching. The typical peak at 1744 cm^−1^ arises from C=O stretching. The band at 1466 cm^−1^ is attributed to C-H bending while other signals at 1163 and 723 cm^−1^ are associated with O=C-O-C stretching and CH_2_ rocking mode respectively. The intensity of all the bands decreased after treatment of the oil with activated clays. The decrease in the intensity of the bands may either be related to removal of pigments only or removal of pigments with some part of the oil (Ramadan and Morsel, 2002). The physical appearance of the Niger oil before and after treatment with 25 % sulfuric acid activated clay has significant difference, and become aesthetically attractive.

## 4 Conclusion

Chemical compositions of the three clay minerals found in different areas of Ethiopia (Zemero, Seladengay, and Mehal Meda) were determined. All the three clay minerals consisted of major oxides. The chemical composition analysis revealed that Zemero clay had high amount of silica content compared to the other two clay minerals, which might suggest its high potential for adsorption capacity to remove coloring substance and impurities from oils. The highest performance in the bleaching of Niger oil (94.5 %) was recorded for the clay mineral collected from Zemero. The other two clays also showed modest performance. In general the result indicates that the activated Zemero clay is an effective, low-cost, easily available and alternative bleaching agent for the removal of coloring pigments and impurities from oil because of its significant bleaching capacity.

## Declarations

### Author contribution statement

Jemal Mohammed Yassin, Yoseph Shiferaw: Conceived and designed the experiments; Performed the experiments; Analyzed and interpreted the data; Contributed reagents, materials, analysis tools or data; Wrote the paper.

Abebe Tedla: Conceived and designed the experiments; Analyzed and interpreted the data; Contributed reagents, materials, analysis tools or data; Wrote the paper.

### Funding statement

This work was supported by Debre Berhan University.

### Data availability statement

Data will be made available on request.

### Declaration of interests statement

The authors declare no conflict of interest.

### Additional information

No additional information is available for this paper.
